# Differences in allergen‐specific basophil activation and T cell proliferation in atopic dermatitis patients with comorbid allergic rhinoconjunctivitis treated with a monoclonal anti‐IL‐4R*α* antibody or allergen‐specific immunotherapy

**DOI:** 10.1002/iid3.808

**Published:** 2023-04-12

**Authors:** Anne‐Sophie Layritz, Jorge Galicia‐Carreón, Said Benfadal, Natalija Novak

**Affiliations:** ^1^ Department of Dermatology and Allergy University Hospital Bonn Bonn Germany

**Keywords:** allergic rhinoconjunctivitis, atopic dermatitis, basophil activation test, T cell proliferation

## Abstract

**Background:**

Atopic dermatitis (AD), a chronic inflammatory disorder, is often accompanied by allergic rhinoconjunctivitis (ARC) as a co‐morbidity. The use of a monoclonal anti‐IL‐4R*α* antibody has been effective in controlling moderate to severe AD symptoms. Allergen‐specific immunotherapy (AIT) is widely used for the treatment of ARC and asthma. The effects of AIT on basophil reactivity/effector functions have already been examined and used as indicators of the treatment efficacy. However, it is unclear, how an anti‐IL‐4R*α* antibody can influence allergen‐specific immune responses of basophils and T cells of AD patients with comorbid ARC.

**Objective:**

To investigate the effect of a monoclonal anti‐IL‐4R*α* antibody on the in vitro allergic responses of basophils and T cells deriving from AD patients with comorbid ARC.

**Methods:**

Blood samples of 32 AD patients were obtained before, after 4 and 16 weeks of an anti‐IL‐4R*α* antibody therapy (300 mg subcutaneously/2 weeks; *n* = 21) or AIT (daily sublingual application; *n* = 11). Patients treated with an anti‐IL‐4R*α* antibody were grouped according to their serum specific immunoglobulin E levels and ARC symptoms, while patients receiving an AIT were additionally grouped according to the allergen specificity of their AIT. Basophil activation test and T cell proliferation assays were undertaken after an in vitro allergen stimulation.

**Results:**

A significant reduction of the immunoglobulin E levels and the allergen‐specific T cell proliferation was observed in AD patients treated with an anti‐IL‐4R*α ‐*antibody, while the allergen‐specific basophil activation/sensitivity were found to be significantly increased. In patients receiving an AIT, the in vitro allergen‐specific basophil activation and the T cell proliferation were found to be significantly decreased in response to seasonal allergens.

**Conclusions:**

An IL‐4R*α* blockade induced by a monoclonal anti‐IL‐4R*α* antibody leads to an increased activity/sensitivity of early effector cells (such as basophils), in contrast to a decreasing reactivity observed under an AIT. The late‐phase T cell reaction to allergens did not differ between the herein assessed treatments.

## Key messages

- Total‐ and specific IgE levels decreased in AD patients treated with an anti‐IL‐4R*α* antibody during the observational period of 16 weeks, but not in patients treated with AIT.
- Allergen‐specific activation and sensitivity increased significantly after allergen stimulation of basophils of AD patients treated with an anti‐IL‐4R*α* antibody for 16 weeks, but decreased under birch‐specific AIT.
- T cell antigen‐specific response to seasonal allergens was reduced in AD patients treated with an anti‐IL‐4R*α* antibody or AIT.

## Graphical abstract

The purpose of our study was to investigate the effect of a monoclonal anti‐IL‐4Rα antibody on the in vitro allergic response of basophils and T cells deriving from AD patients with comorbid ARC. We conclude, that an IL‐4R*α* blockade induced by a monoclonal anti‐IL‐4R*α*‐antibody leads to an increased activity/sensitivity of early effector cells (such as basophils), in contrast to a decreasing reactivity observed under an AIT. The late‐phase T cell reaction to allergens did not differ between the herein assessed treatments.

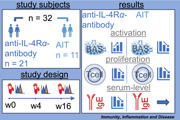

**Abbreviations**
7‐AAD: 7‐amino actinomycin D
AD: atopic dermatitis
AIT: allergen‐specific immunotherapy
ARC: allergic rhinoconjunctivitis
BAT: basophil activation test
CD‐max: basophil maximal reactivity
CD‐sens: basophil allergen threshold sensitivity
CFSE: carboxyfluorescein‐diacetate‐succinimidyl‐ester
CSU: chronic spontaneous urticaria
FCS: fetal calf serum
FcεRI: high‐affinity receptor for the Fc region of immunoglobulin E
fMLP: *N*‐formylmethionyl‐leucyl‐phenylalanine
IL‐13: interleukin‐13
IL‐4: interleukin‐4
IL‐4R*α*
: interleukin‐4 receptor alpha
PBMCs: peripheral blood mononuclear cells
PBS: phosphate buffered saline
rBet v1: *Betula verrucosa*, allergen 1, recombinant
RCAT: Rhinitis Control Assessment Test
rPhl p1: *Phleum pratense*, allergen 1, recombinant
rPhl p5: *Phleum pratense*, allergen 5, recombinant
sIgE: specific immunglobulin‐E
Th2: helper T (cell) type 2
Treg: regulatory T (cell)

## 1 INTRODUCTION

Atopic dermatitis (AD) is a complex, chronic inflammatory skin disease that is characterized by recurrent eczematous lesions and pruritus and that can be complicated by viral or bacterial superinfections.^1^, ^2^, ^3^, ^4^ AD might precede the development of several comorbidities such as allergic rhinoconjunctivitis (ARC), with symptoms such as rhinorrhea, nasal pruritus, sneezing, itching and ocular surface inflammation.^5^, ^6^, ^7^, ^8^, ^9^, ^10^, ^11^, ^12^, ^13^ For patients with moderate to severe AD, a systemic therapy is available through the use of a monoclonal antibody against the interleukin‐4 (IL‐4) receptor alpha (IL‐4R*α*); this anti‐IL‐4R*α* antibody blocks the IL‐4/interleukin‐13 (IL‐13) binding to IL‐4 receptor types 1 and 2.^14^, ^15^, ^16^, ^17^, ^18^ Previous 16‐week‐studies and subsequent clinical trials have shown that the monoclonal anti‐IL‐4R*α* antibody can substantially improve the AD symptoms in adults, adolescents and children between 6 and 11 years of age, with acceptable safety.^19^, ^20^, ^21^

On the other hand, allergen‐specific immunotherapy (AIT) has an immunomodulatory impact on the course of ARC and asthma, as well as a predictive effect on the development/worsening of polysensitisation or of asthma.^6^, ^11^, ^22^, ^23^, ^24^, ^25^, ^26^, ^27^, ^28^, ^29^, ^30^, ^31^ A successful AIT regimen can reduce the activity of effector cells at early timepoints, while at the same time it can induce allergen‐specific CD4+ regulatory T (Treg) cells, support the isotype switching of B cells and increase the release of immunosuppressive mediators such as interleukin‐10, transforming growth factor beta and interleukin‐35 by regulatory B cells and Tregs.^6^, ^15^, ^29^, ^30^, ^31^, ^32^, ^33^, ^34^

Basophil granulocytes represent <1% of the leukocytes in the peripheral blood.^35^, ^36^ Nevertheless, basophils play a key role in immediate type allergic reactions by producing IL‐4 and IL‐13. Through these ILs, basophils induce immunoglobulin E (IgE) synthesis in B cells as well as T cell differentiation,^37^, ^38^ and therefore, they maintain reactions through the adaptive immune system.^37^ The basophil activation test (BAT; performed with surface markers CD203c and CD63) is reliable, validated and widely used for the diagnostic evaluation of the basophil maximal reactivity (CD‐max).^39^ The basophil allergen threshold sensitivity (CD‐sens), defined as the effective dose at 50% (EC50) of CD‐max, might be an effective tool for the monitoring of allergen‐specific immunotherapies.^39^, ^40^

While the effects of AIT on basophils have already been examined in several studies,^40^, ^41^, ^42^, ^43^, ^44^ it is still unclear how a monoclonal anti‐IL‐4R*α* antibody influences the allergen‐specific immune responses of early effector cells and T cells in patients with AD and comorbid ARC.

This study has aimed at evaluating the effect of a monoclonal anti‐IL‐4R*α* antibody on the total and specific IgE (sIgE) serum levels, the in vitro basophil activation and the T cell allergen proliferation in individuals bearing AD and ARC symptoms and being diagnosed based on the serum allergen sIgE for common birch or timothy grass.

## 2 METHODS

### 2.1 Study design and study population

This prospective, single center study took place at the Department of Dermatology and Allergy of the University Hospital Bonn from 2019 until 2021. Patients were included according to the following inclusion criteria: age ≥18 and active diagnosis of AD and comorbid ARC.

AD was diagnosed based on the criteria of Hanifin and Rajka^45^ and on the UK working party diagnostic criteria.^46^, ^47^ ARC was diagnosed based on the patient symptom history, the Rhinitis Control Assessment Test (RCAT ≤ 17), and sIgE (≥ 5 kU/L). Exclusion criteria were comorbidities such as malignomas, immunodeficiency, severe chronic diseases and pregnancy. All donors provided their written informed consent before the beginning of the study. The study followed the ethical principles of the Declaration of Helsinki and was approved by the local Ethics Committee (approval number: 267/19).

Initially, 44 patients were enrolled. Five patients discontinued the study protocol for personal reasons. In seven patients, the clinical parameters of the ARC symptoms based on the RCAT did not match with the sIgE levels of their birch pollen and grass pollen senitization, so we were not able to determine with certainty if the ARC symptoms were related to the seasonal allergens of birch pollen (rBet v1; *Betula verrucosa*, allergen 1, recombinant) or timothy grass pollen (rPhl p1 or p5; *Phleum pratense*, allergen 1 or 5, recombinant).

Two observation groups were formed: the index group and the control group. The index group included 21 patients (13 females and 8 males; age range: 18–69 years; median age: 29 years) with AD and comorbid ARC that received an anti‐IL‐4R*α* antibody therapy (demographic data presented in Table 1). All patients treated with the monoclonal anti‐IL‐4R*α* antibody were grouped according to their exposure to rBet v1, rPhl p1/rPhl p5, as well as according to their ARC symptoms (based on the RCAT). The control group consisted of 11 patients (seven females and four males; age range: 24−59 years; median age: 31 years) with AD and comorbid ARC that were treated with daily sublingual AIT (demographic data presented in Table 1). Patients treated with AIT were grouped according to their sIgE levels, their ARC symptoms and their allergen‐specific treatment for birch and grass pollen.

**Table 1:** Demographics and characteristics of the study population.

	Anti‐IL‐4R*α* antibody	AIT
Timepoint	w0	w0
Sample size	*n* = 21	*n* = 11
Age [MD (range)]	29 (18–69)	32 (24–59)
Gender: female	13	7
Gender: male	8	4
ARC symptoms based on the RCAT (MD ± *SD*)	17.00 ± 5.30	20.00 ± 4.52
Log total IgE in IU/mL (MD ± *SD*)	3.25 ± 0.84	3.12 ± 0.67
Log rBet v1 in kU/L (MD ± *SD*)	1.74 ± 0.54	1.78 ± 0.43
Log rPhl p1 in kU/L (MD ± *SD*)	1.28 ± 0.96	0.14 ± 0.66

*Note*: The sample size, age (MD and range), gender, RCAT (MD ± *SD*), log total IgE (MD ± *SD*), and log sIgE (ret v1; rPhl p1) (MD ± *SD*) are given for the observational timepoint w0 (week 0 = before therapy), in the two therapy groups of an anti‐IL‐4R*α* antibody treatment and AIT.

Abbreviations: AIT, allergen‐specific immunotherapy; anti‐IL‐4R*α* antibody, anti interleukin‐4 receptor alpha antibody; MD, median; RCAT, Rhinitis Control Assessment Test; *SD*, standard deviation; w0, week 0 = before therapy.

To classify the observable effects of the monoclonal anti‐IL‐4R*α* antibody on allergen‐specific immune responses, we selected AD patients with AIT‐treated ARC as a control group, since the effects of AIT on the IgE levels and the effector cells have already been examined in previous studies.^40^, ^41^, ^42^, ^43^, ^44^

The observational time was 16 weeks, based on data from a registration study of a monoclonal anti‐IL‐4R*α* antibody,^18^ in which the clinical parameters associated with skin lesions and the quality of life were shown to improve significantly during this period. Moreover, significant changes could be indicated more easily at the beginning of the treatment without disruptive factors or external triggers.

The observational period was assessed at three timepoints: (i) before the start of any systemic treatment (w0), (ii) at 4 weeks (w4) after the start of a systemic treatment and (iii) at 16 weeks (w16) after the start of a systemic treatment. All patients filled in a questionnaire based on the Dermatology Life Quality Index (DLQI), the RCAT and the Eczema Area and Severity Index (EASI); the answers to this questionnaire were evaluated to assess the efficacy of the treatment, to ensure the correct handling and to detect nonresponders. The DLQI was used so as to estimate the effects of skin diseases on daily life routine and their impact on the quality of life.^48^, ^49^ Additionally, questions based on the RCAT were used to objectify the ARC symptoms, while the EASI was raised so as to classify the expression and severity of AD.^48^

Blood samples were obtained (by using Serum Monovette; 7.5 mL; Sarstedt AG & Co KG) from patients to determine the total IgE serum levels through an electrochemiluminescence immunoassay (by using Cobas® e801; Roche Diagnostics)^50^ and the sIgE serum levels for birch (rBet v1) and grass (rPhl p1, rPhl p5) pollen through a fluoroenzyme immunoassay (by Phadia 250; Thermo Fisher Scientific, Phadia AB),^51^ the activation level of basophils^52^ and the T lymphocyte proliferation. Carboxyfluorescein‐diacetate‐succinimidyl‐ester (CFSE) T cell allergen proliferation assays were evaluated in 11 donors treated with a monoclonal anti‐IL‐4R*α* antibody and in 10 patients treated with AIT. Proliferation assays were performed in individuals sensitized to rBet v1 or rPhl p1/rPhl p5.

### 2.2 BAT

**Figure 1:**
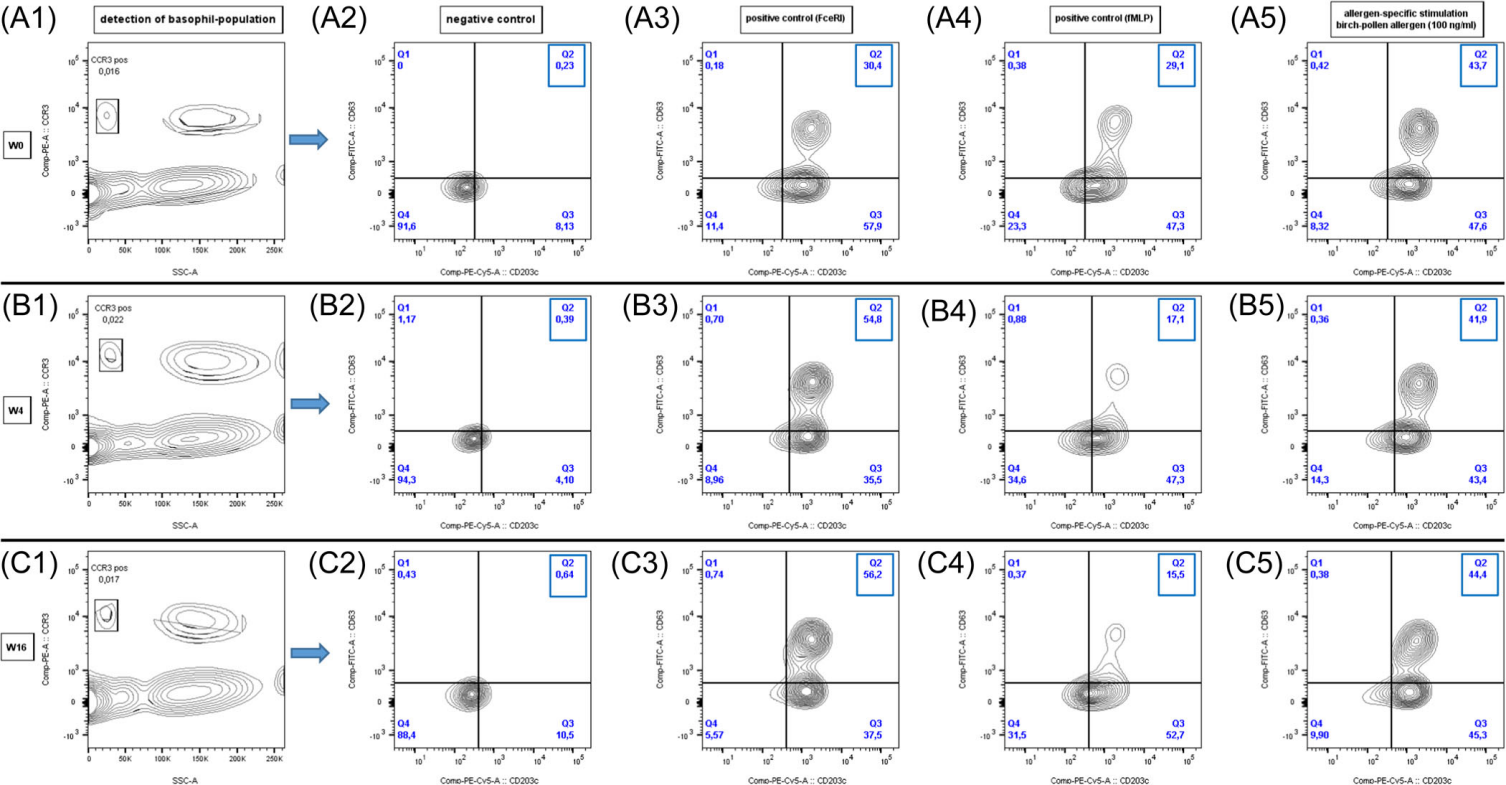
An example of a gating strategy and representative data of the CD203c+/CD63+ basophils for three timepoints [week 0 (w0), week 4 (w4), and week 16 (w16)]; [A1 (w0), B1 (w4), C1 (w16)] detection of basophils: The basophil population was gated as CCR3+ cells (y‐axis) and SSC^low^ (x‐axis); [A2 (w0), B2 (w4), C2 (w16)] negative control: The detected cells were displayed in one graph with CD63‐FITC (y‐axis) and CCR‐PE (x‐axis) and one graph with CD203c‐PE‐DY647 (y‐axis) and CCR‐PE (x‐axis). In A2 (w0), B2 (w4), C2 (w16) both markers were set in one graph. For the negative control (basophil stimulation with a cellular buffer) the quadrant gate was set for an activation level <5% (Q2); [A3 (w0), B3 (w4), C3 (w16)] positive controls: The same quadrant position as set for the negative control was used for the positive controls via Fc*ε*RI [A3 (w0), B3 (w4), C3 (w16)] and fMLP [A4 (w0), B4 (w4), C4 (w16)] and allergen‐specific stimulation [A5 (w0), B5 (w4), C5 (w16)]; an example of allergen‐specific basophil stimulation with birch pollen allergen (dilution 1:100 ng/mL) during an anti‐IL‐4R*α* antibody treatment is shown in A5 (w0), B5 (w4), C5 (w16).

EDTA‐blood (collected in a 9‐mL tube using S‐Monovette; Sarstedt AG & Co KG) was used to verify the activation of basophil cells via the detection of surface markers (CD203c and CD63) through the undertaking of BAT according to the standardized instruction manual (Bühlmann Laboratories AG).^52^ Data were acquired by using a flow cytometer emitting at 488 nm (FACSCanto; Becton Dickinson GmbH). An example of the gating strategy and the allergen‐specific basophil activation is visualized in Figure 1A‐C, as generated by FlowJo version 10.8.0.^53^

In an attempt to ensure the high quality of the undertaken BAT, the three typical leukocyte populations (i.e., lymphocytes, monocytes and granulocytes) had to be detected in the blood samples before gating as a criterion of blood samples' quality. The absolute number of basophils ranged between 200 and 600 and the percentage of basophils in the negative control was determined as <5%.^52^ BAT assays were performed within the first 24 h after the blood collection, according to the standardized instruction manual (Bühlmann Laboratories AG).^52^ If the blood samples had to be stored, they were kept protected from light and refrigerated at 2°C–8°C.

### 2.3 Isolation of peripheral blood mononuclear cells (PBMCs)

Peripheral blood was collected into sodium heparin tubes (9‐mL Heparin S‐Monovette; Starstedt AG & Co KG) and PBMCs were isolated with the use of a Lymphoprep™ (Sigma‐Aldrich) density gradient after centrifugation at 2100 rpm, for 30 min, at room temperature (25°C). After centrifugation, the mononuclear cell layer was collected, washed twice and counted by using an automated cell counter (NucleoCounter NC‐200; ChemoMetec).

### 2.4 Allergens

The undertaken BAT and antigen‐specific T cell proliferation assay were induced by freeze‐dried allergens obtained by Bühlmann Laboratories AG (Schönenbuch, Switzerland). Each allergen was prepared as an aqueous extract in 250 μL of stimulation buffer, until the extract was dissolved. The allergens for BAT were used at different dilutions to rule out any dose‐dependent effects (1 = 100 ng/mL; 1:1 = 50 ng/mL, 1:10 = 10 ng/mL, and 1:100 = 1 ng/mL).

### 2.5 CFSE labeling

CFSE mixed isomers (Vybrant CFDA‐SE Cell Tracer Kit; Invitrogen) were used for the CFSE labeling. In brief, CFSE was stored frozen as a 10‐mM stock solution until used. A pellet of 1 × 10^6^ cells was resuspended in 1 mL with 1% fetal calf serum (FCS)–phosphate buffered saline (PBS) solution and a CFSE labeling solution of a concentration ranging from 0.5 to 10 μM to 37 nM and were incubated for 8 min, at room temperature, in the dark. After incubation, 1 mL of FCS per 2 × 10^6^ cells was added to each sample, followed by a 10min incubation. Cells were then washed twice with a 2% FCS‐PBS solution and were centrifuged at 1350 rpm for 10 min, before resuspension in RPMI.^54^ PBMCs stained with CFSE were analysed before and after 7 days of cell culturing, through flow cytometry (FACSCanto; Becton Dickinson GmbH). Analysis was performed by using the BD FACSDiva software version 5.0.3 and the FlowJo version 10.8.0 for Mac OS X software.^53^, ^55^

### 2.6 Cell viability determination

After the CFSE labeling, cells were stained with 7‐amino actinomycin D (7‐AAD; 0.2 μg/10^6^ cells) (Sigma‐Aldrich) and were incubated for 5 min before their analysis through flow cytometry.^54^ 7‐AAD‐positive cells were considered as dead and discarded for T cell proliferation analysis.

### 2.7 Cell culture

PBMCs were cultured in 96‐well flat‐bottomed plates (Corning Inc.) at a density of 0.2 × 10^6^ cells/well, in RPMI‐1640 medium supplemented with 1 mM sodium pyruvate, 2 mM l‐glutamine, 50 μg/mL gentamicin and 10% heat‐inactivated FCS. Cells were incubated at 37°C, in a 5% CO_2_ humidified chamber. Antigen‐specific stimulation was performed by adding the corresponding allergen in diluted concentrations (1 = 100 ng/mL, 1:1 = 50 ng/mL, 1:10 = 10 ng/mL, and 1:100 = 1 ng/mL). Likewise, the cells were co‐stimulated at day 0 in the presence or absence of MACSiBead™ (4 × 10^8^ particles) being conjugated to the monoclonal anti‐biotin human antibodies against CD2 (CD2‐biotin; 100 μg/mL), CD3 (CD3‐biotin; 100 μg/mL) and CD28 (CD28‐biotin; 100 μg/mL) (T Cell Activation/Expansion Kit, human; Miltenyi Biotec). Five experimental conditions were analysed to determine the T cell proliferation index after the in vitro allergen stimulation in both groups of donors. CFSE‐labeled cells were cultured for 7 days with the corresponding allergen and were co‐stimulated with MACSiBeads™ (at a ratio of 1:10 per 1 × 10^6^ cells) and with of recombinant human IL‐2 (PeproTech; at a ratio of 50−100 UI/mL per 1 × 10^6^ cells, every 2 days). Moreover, CFSE‐labeled PBMCs were cultured for 10 days with human recombinant IL‐2 (50−100 UI/mL, every 48 h) and the related allergen dilutions. Additionally, cells were cultured only with monoclonal anti‐biotin human antibodies against CD2, CD3, and CD28 (100 μg/mL) and with IL‐2 (50−100 UI/mL per 1 × 10^6^ cells, every 2 days) and they were harvested after 7 and 10 days of culturing to serve as cell stimulation positive controls. Furthermore, cells were cultured only with IL‐2 (50–100) and without any costimulation (RPMI). The fluorescence of the stimulated CD4+ CFSE‐diluted cells was measured after 7 and 10 days of cell culturing, through flow cytometry.

### 2.8 T cell proliferation assay

**Figure 2:**
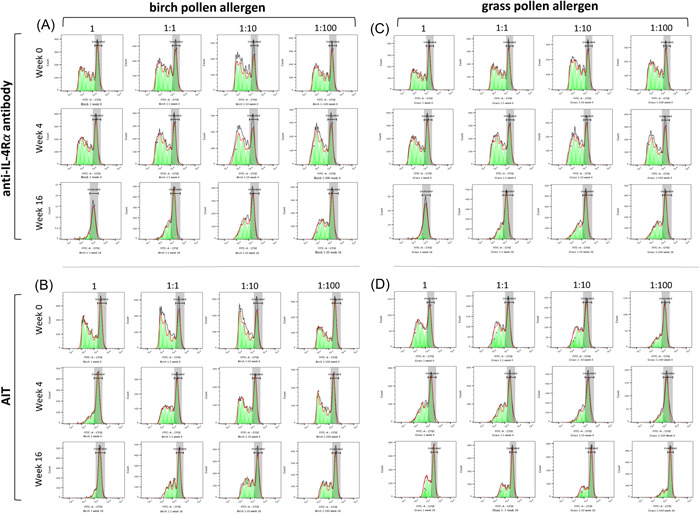
Representative histograms of T cell proliferation. Representative histograms showing a progressive dilution in the CFSE fluorescent intensity of proliferating T cells for anti‐IL‐4R*α* antibody (A, C) and AIT (B, D) treated AD patients. For the assays, PBMC (1 × 10^6^ cells) were incubated with CFSE (0.5 μM) at room temperature for 8 min and the reaction was blocked by addition of FCS. The cells were washed in 2% FCS‐PBS solution. Afterwards, the cells were cultured and stimulated with birch‐ and grass pollen allergen. The fluorescence of the stimulated CD4+ CFSE‐diluted cells was measured after 7 days using flow cytometry. T cell proliferation of CD4+ CFSE+ T cells of AD patients was analysed after 7 days of allergen stimulation with birch pollen allergen (A, B) and grass pollen allergen (C, D) at dilutions 1 (100 ng/mL), 1:1 (50 ng/mL), 1:10 (10 ng/mL) and 1:100 (1 ng/mL) at week 0 (w0), 4 (w4) and 16 (w16), respectively. AD, atopic dermatitis; FCS, fetal calf serum; PBMC, peripheral blood mononuclear cells.

T lymphocyte proliferation was monitored by establishing a dose‐response curve with the culprit allergen. Therefore, a dose‐response curve using four allergen concentrations (1 = 100 ng/mL; 1:1 = 50 ng/mL, 1:10 = 10 ng/mL, and 1:100 = 1 ng/mL) was established after 7 days of cell culturing. T cell proliferation was evaluated through the undertaking of flow cytometry and the obtained data were analysed with the use of FlowJo version 10.8.0.^53^ Representative histograms of the allergen‐specific T cell proliferation are presented in Figure 2A−D and Figure S1a−d.

T cell proliferation assays were analysed through the calculation of the average number of divisions that all responding cells have undergone since the initiation of the culturing process (proliferation index). Similarly, the cell growth was evaluated by calculating the number of divisions for all cells in the original culture (division index) versus the number of undivided cells after 7 days of allergen stimulation.^56^

### 2.9 Statistics

All collected data were analysed with one‐way analysis of variance (ANOVA) followed by Tukey's multiple comparison test by using GraphPad Prism version 9.1.2 for MacOS. Differences were considered as statistically significant when the test yielded *p* values that were <.05.

For the analysis of the index group (treated with the anti‐IL‐4R*α* antibody; *n* = 21), patients with ARC symptoms exhibiting ≤17 score points (based on the RCAT) and/or sIgE serum levels ≥5 kU/L were included. For the analysis of the control group (receiving an AIT; *n* = 11), seven patients receiving a birch pollen‐specific AIT and four patients receiving a grass pollen‐specific AIT were analysed. For patients that dropped out or for those whose diagnosis of seasonal‐related ARC was not ensured, their data were excluded. Finally, 32 patients were included into the statistical analysis.

For the BAT analysis the calculation of the CD‐sens was based on a dose‐response curve adapted from Hoffmann et al.^39^ and Santos et al.^57^ The data were analysed with one‐way ANOVA followed by a Friedman test.

The results of the proliferative capacity of responding T cells are shown in units deriving from the division index parameter as the total number of divisions per number of cells at the beginning of the culturing process.

The figures were generated through GraphPad Prism version 9.1.2. and FlowJo version 10.8.0.^53^

## 3 RESULTS

### 3.1 Decrease in the total IgE and sIgE serum levels was observed in patients treated with an anti‐IL‐4R*α* antibody, but not in patients receiving an AIT

**Figure 3:**
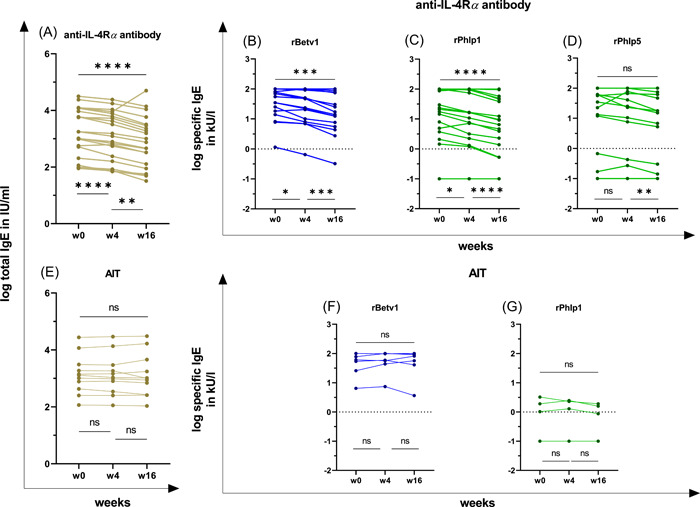
Total IgE in IU/mL and specific IgE in kU/L in patients receiving an anti‐IL‐4R*α* antibody or AIT, before therapy (w0) and at week 4 (w4) and week 16 (w16); logarithmic data: log10; ns nonsignificant; *p < .05; **p < .01; ***p < .001; ****p < .0001. (A, E) total IgE. (A) anti‐IL‐4R*α* antibody: *n* = 21, *p* (w0–w4) <.0001; *p* (w0–w16) <.0001; *p* (w4–w16) <.01; (E) AIT: *n* = 11. (B–D; F, G) Specific IgE. (B–D) Anti‐IL‐4R*α* antibody: rBetv1: *n* = 17, *p* (w0–w4) <.05; *p* (w0–w16) <.001; *p* (w4–w16) <.001; rPhlp1: *n* = 18, *p* (w0–w4) <.05; *p* (w0–w16) <.0001; *p* (w4–w16) <.0001; rPhlp5: *n* = 16, *p* (w4–w16) <.01; (F–G) AIT: rBetv1: *n* = 7; rPhlp1: *n* = 4.

The levels of the total IgE decreased significantly after 4 and 16 weeks in patients treated with an anti‐IL‐4R*α* antibody (w0–w4: *p* < .0001; w0–w16: *p* < .0001; w4–w16: *p* < .01; Table 2A and Figure 3A). Additionally, the serum levels of sIgE decreased significantly after 4 and 16 weeks under an anti‐IL‐4R*α* antibody treatment (**rBet v1**: w0–w4, *p* < .05; w0–w16, *p* < .001; w4–w16, *p* < .001; **rPhl p1**: w0–w4, *p* < .05; w0–w16, *p* < .0001; w4–w16, *p* < .0001; **rPhl p5**: w4–w16, *p* < .01; Table 2A and Figure 3B−D). In contrast, the levels of total IgE and sIgE did not change significantly until week 16 in patients receiving an AIT (Table 2H and Figure 3E−G).

**Table 2:** Median, IQR, and *p* values for the parameters IgE, basophil activation and sensitivity (EC‐50, CD‐sens), division index for T cell proliferation and ARC symptoms based on the RCAT.

Anti‐IL‐4R*α* antibody	MD w0	MD w4	MD w16	IQR w0	IQR w4	IQR w16	*p* (w0–w4)	*p* (w0–w16)	*p* (w4–w16)
A—log(10) total/specific IgE									
log(10) total IgE in IU/mL (*n* = 21)	3.25	3.09	2.88	2.50–4.00	2.30–3.80	2.00–3.50	****	****	**
log(10) sIgE (rBetv1) in kU/L (*n* = 17)	1.74	1.67	1.23	1.20–2.00	0.95–2.00	0.81–1.92	*	***	***
log(10) sIgE (rPhlp1) in kU/L (*n* = 18)	1.28	1.12	0.89	0.51–1.99	0.29–1.98	0.04–1.67	*	****	****
log(10) sIgE (rPhlp5) in kU/L (*n* = 16)	1.10	0.95	0.78	(−1.00)–1.77	(−1.00)–1.77	(−1.00)–1.57	ns	ns	**
B—BAT Birch (*n* = 17) in %									
% CD203c+/CD63+ dilution 1 (100 ng/mL)	52.60	55.50	59.50	42.20–65.00	35.70–69.05	52.25–63.15	ns	ns	ns
% CD203c+/CD63+ dilution 1.1 (50 ng/mL)	61.20	60.50	63.60	41.00–69.20	38.90–66.85	52.95–71.50	ns	*	ns
% CD203c+/CD63+ dilution 1.10 (10 ng/mL)	51.50	64.50	57.50	15.55–61.95	13.30–71.65	25.90–72.70	ns	***	ns
% CD203c+/CD63+ dilution 1.100 (1 ng/mL)	2.00	0.80	1.90	0.05–9.65	0.00–14.35	0.50–23.45	ns	ns	ns
EC50 in ng/mL	0.42	0.21	0.28	0.15–1.78	0.06–0.95	0.07–0.66	ns	*	ns
CD‐sens	235.40	510.00	369.80	57.77–954.80	107.20–1590.00	152.70–1471.00	ns	*	ns
C—BAT Grass (*n* = 19) in %									
% CD203c+/CD63+ dilution 1 (100 ng/mL)	53.40	66.70	64.30	46.10–72.50	39.80–78.50	47.50–68.60	ns	ns	ns
% CD203c+/CD63+ dilution 1.1 (50 ng/mL)	59.40	60.90	64.30	43.40–73.70	30.10–78.80	45.80–71.50	ns	ns	ns
% CD203c+/CD63+ dilution 1.10 (10 ng/mL)	55.10	61.30	66.30	23.40–63.80	9.60–74.70	37.90–74.70	ns	**	ns
% CD203c+/CD63+ dilution 1.100 (1 ng/mL)	3.30	3.50	14.30	0.00–13.50	0.20–23.40	0.50–32.30	ns	**	ns
EC50 in ng/mL	0.37	0.31	0.07	0.26–0.43	0.08–0.43	0.05–0.38	ns	***	**
CD‐sens	266.70	322.30	1449.00	230.80–381.50	230.00–1204.00	263.10–1967.00	ns	***	**
D—positive controls BAT in %									
fMLP	39.60	32.00	20.00	17.25–56.25	12.45–55.35	12.90–53.10	ns	*	ns
Fc*ε*RI	59.90	64.90	62.40	47.00–69.50	51.60–73.95	51.15–72.15	ns	ns	ns
E—CFSE Birch (*n* = 11) division index									
CD4+ T‐cells dilution 1 (100 ng/mL)	0.53	0.55	0.40	0.26–0.68	0.31–0.64	0.32–0.65	ns	ns	ns
CD4+ T‐cells dilution 1.1 (50 ng/mL)	0.60	0.59	0.50	0.53–0.74	0.37–0.73	0.36–0.67	ns	ns	ns
CD4+ T‐cells dilution 1.10 (10 ng/mL)	0.70	0.63	0.55	0.61–0.73	0.38–0.70	0.39–0.74	ns	*	ns
CD4+ T‐cells dilution 1.100 (1 ng/mL)	0.65	0.58	0.47	0.51–0.76	0.36–0.65	0.37–0.59	ns	**	ns
F—CFSE Grass (*n* = 11) division index									
CD4+ T‐cells dilution 1 (100 ng/mL)	0.61	0.55	0.36	0.42–0.72	0.28–0.62	0.26–0.50	ns	ns	ns
CD4+ T‐cells dilution 1.1 (50 ng/mL)	0.56	0.57	0.40	0.47–0.72	0.27–0.65	0.28–0.51	ns	*	ns
CD4+ T‐cells dilution 1.10 (10 ng/mL)	0.60	0.53	0.43	0.54–0.67	0.28–0.66	0.30–0.49	ns	**	ns
CD4+ T‐cells dilution 1.100 (1 ng/mL)	0.57	0.46	0.38	0.50–0.63	0.21–0.64	0.14–0.49	ns	*	ns
G—ARC symptoms based on RCAT (*n* = 21)									
RCAT	17.00	19.00	17.00	11.00–19.00	17.00–21.00	14.00–20.50	*	ns	ns
AIT									
H—log(10) total/specific IgE									
log(10) total IgE in IU/mL (*n* = 11)	3.11	3.03	3.00	2.63–3.49	2.54–3.47	2.43–3.66	ns	ns	ns
log(10) sIgE (rBetv1) in kU/L (*n* = 7)	1.78	1.77	1.91	1.41–1.90	1.64–2.00	1.61–1.97	ns	ns	ns
log(10) sIgE (rPhlp1) in kU/L (*n* = 4)	0.15	0.24	0.07	(−0.75)–0.45	(−0.72)–0.38	(−0.77)–0.26	ns	ns	ns
I—BAT Birch (*n* = 7) in %									
% CD203c+/CD63+ dilution 1 (100 ng/mL)	65.80	54.30	65.40	52.90–74.00	44.10–76.60	45.50–66.40	ns	*	ns
% CD203c+/CD63+ dilution 1.1 (50 ng/mL)	72.40	60.10	62.40	49.70–75.80	50.00–72.70	53.90–66.60	ns	ns	ns
% CD203c+/CD63+ dilution 1.10 (10 ng/mL)	36.60	38.00	27.50	12.80–75.40	24.30–71.60	15.90–61.00	ns	ns	ns
% CD203c+/CD63+ dilution 1.100 (1 ng/mL)	0.90	0.30	1.00	0.00–20.00	0.00–15.40	0.30–1.20	ns	ns	ns
EC50 in ng/mL	0.44	0.50	0.52	0.07–0.79	0.14–0.63	0.33–1.41	ns	ns	ns
CD‐sens	226.60	201.90	194.00	128.50–1541.00	159.80–1186.00	71.02–303.10	ns	ns	ns
J—BAT Grass (*n* = 4) in %									
% CD203c+/CD63+ dilution 1 (100 ng/mL)	34.95	39.70	29.90	8.50–45.95	5.93–59.30	5.95–55.43	ns	ns	ns
% CD203c+/CD63+ dilution 1.1 (50 ng/mL)	22.75	28.15	23.70	4.40–35.40	6.08–42.80	1.28–45.68	ns	ns	ns
% CD203c+/CD63+ dilution 1.10 (10 ng/mL)	2.55	4.60	3.50	0.08–51.23	1.00–5.65	0.45–8.05	ns	ns	ns
% CD203c+/CD63+ dilution 1.100 (1 ng/mL)	0.50	0.05	0.45	0.125–0.65	0.00–2.13	0.00–2.25	ns	ns	ns
EC50 in ng/mL	1.42	1.42	1.44	0.19–2.54	0.93–2.50	0.88–5.24	ns	ns	ns
CD‐sens	136.40	74.91	70.02	39.45–740.80	41.47–108.90	27.49–120.70	ns	ns	ns
K—positive controls BAT in %									
fMLP	21.80	26.10	22.40	9.00–37.30	13.70–29.30	12.10–26.20	ns	ns	ns
Fc*ε*RI	66.70	62.60	63.20	59.60–79.30	54.50–80.30	41.50–72.40	ns	ns	ns
L—CFSE Birch (*n* = 5) division index									
CD4+ T‐cells dilution 1 (100 ng/mL)	0.44	0.49	0.29	0.32–0.54	0.24–0.55	0.18–0.34	ns	ns	ns
CD4+ T‐cells dilution 1.1 (50 ng/mL)	0.56	0.41	0.36	0.50–0.68	0.33–0.63	0.31–0.44	ns	ns	ns
CD4+ T‐cells dilution 1.10 (10 ng/mL)	0.56	0.47	0.40	0.50–0.60	0.35–0.66	0.32–0.52	ns	ns	ns
CD4+ T‐cells dilution 1.100 (1 ng/mL)	0.48	0.45	0.35	0.41–0.51	0.35–0.55	0.21–0.46	ns	ns	ns
M—CFSE Grass (*n* = 5) division index									
CD4+ T‐cells dilution 1 (100 ng/mL)	0.41	0.38	0.31	0.30–0.64	0.25–0.45	0.30–0.37	ns	ns	ns
CD4+ T‐cells dilution 1.1 (50 ng/mL)	0.43	0.43	0.34	0.38–0.62	0.36–0.49	0.27–0.41	ns	ns	**
CD4+ T‐cells dilution 1.10 (10 ng/mL)	0.43	0.43	0.39	0.33–0.61	0.33–0.53	0.23–0.43	ns	ns	ns
CD4+ T‐cells dilution 1.100 (1 ng/mL)	0.32	0.41	0.25	0.23–0.51	0.24–0.43	0.14–0.35	ns	ns	ns
N—ARC symptoms based on RCAT (*n* = 11)									
RCAT	20.00	21.00	19.00	12.00–22.00	18.00–23.00	13.00–20.00	ns	ns	ns

*Note*: The median, the IQR and the *p* values for IgE, basophil activation and sensitivity (EC‐50/CD‐Sens), division index of T cell proliferation and ARC symptoms based on the RCAT are given for the three observational timepoints w0 (week 0), w4 (week 4), and w16 (week 16), in two therapy groups (A–G: anti‐IL‐4Rα antibody; H–N: AIT); analysed by one‐way ANOVA Tukey's multiple comparison test and Friedman‐test.

Abbreviations: ANOVA, analysis of variance; anti‐IL‐4R*α* antibody, anti interleukin‐4 receptor alpha antibody; BAT, basophil activation test; CD‐sens, basophil allergen threshold sensitivity; CFSE, 5‐(und‐6)‐carboxyfluorescein‐diacetate‐succinimidyl‐ester; EC‐50, effective concentration (dose) at 50% of the basophil reactivity; Fc*ε*RI, Fc‐epsilon‐receptor‐1‐alpha; fMLP, N‐formyl‐methionyl‐leucyl‐phenylalanine; IQR, interquartile range; MD, median; ns, nonsignificant; *p*, *p* value; RCAT, Rhinitis Control Assessment Test; w0, week 0; w4, week 4; w16, week 16.

* *p* < .05

** *p* < .01

*** *p* < .001

**** *p* < .0001.

### 3.2 Allergen‐specific basophil activation and sensitivity increased in patients treated with a monoclonal anti‐IL‐4R*α* antibody, but decreased in patients under birch pollen‐specific AIT

To investigate the effects of a monoclonal anti‐IL‐4R*α* antibody and of an AIT on early effector cells, we analysed the in vitro activation response on CD203c + /CD63 + basophils at four different allergen concentrations (1 = 100 ng/mL, 1:1 = 50 ng/mL, 1:10 = 10 ng/mL, and 1:100 = 1 ng/mL). In an attempt to detect dose‐dependent effects, four different allergen dilutions were used for the induction of allergen stimulation. We calculated the basophil sensitivity (CD‐sens = 1/EC50 × 100) based on a dose‐response curve adapted from Hoffmann et al.^39^ and Santos et al.^57^

In patients treated with the monoclonal anti‐IL‐4R*α* antibody and sensitized toward rBet v1, the in vitro basophil activation in response to the birch pollen allergen increased significantly depending on the allergen concentration.

**Figure 4:**
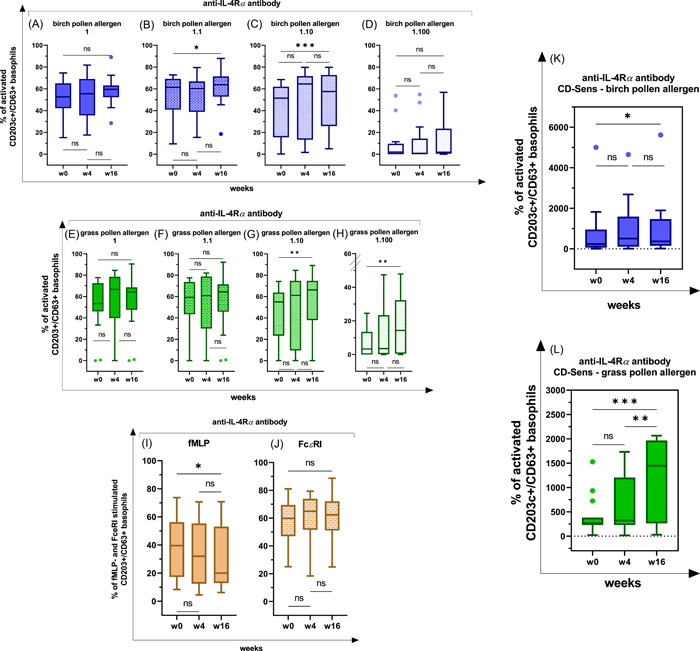
Basophil reactivity and sensitivity in AD patients with senitization for birch‐ and grass pollen, receiving an anti‐IL‐4R*α* antibody; BAT was performed with four different allergen concentrations (1 = 100 ng/mL, 1:1 = 50 ng/mL, 1:10 = 10 ng/mL, 1:100 = 1 ng/mL) for three timepoints [before therapy (w0) and at week 4 (w4) and week 16 (w16)]; ns nonsignificant; *p < .05; **p < .01; ***p < .001; ****p < .0001. In the box‐whisker plots the median, 25th and 75th percentile, the range and outliers are represented. (A–D) Birch pollen specific stimulation: *n* = 17, dilution level 1 (100 ng/mL): *p* (w0–w16) = ns; dilution level 1:1 (50 ng/mL): *p* (w0–w16) <.05; dilution level 1:10 (10 ng/mL): *p* (w0–w16) <.001; dilution level 1:100 (1 ng/mL) *p* = ns; (E–H) grass pollen specific stimulation: *n* = 19, dilution level 1 (100 ng/mL): *p* = ns; dilution level 1:1 (50 ng/mL): *p* = ns; dilution level 1:10 (10 ng/mL): *p* (w0–w16) <.01; dilution level 1:100 (1 ng/mL): *p* (w0–w16) <.01; (I) fMLP‐mediated stimulation: *n* = 21, *p* (w0–w16) <.05; (J) Fc*ε*RI‐mediated stimulation: *n* = 21, *p* = ns; (K) CD‐sens in birch pollen stimulated CD203c+/CD63+ basophils, *p* (w0–w16) <.05; (L) CD‐sens in grass pollen stimulated CD203c+/CD63+ basophils, *p* (w0–w16) <.001, *p* (w4–w16) <.01. AD, atopic dermatitis.

At allergen dilution 1 (100 ng/mL) no significant changes were observed after 16 weeks of an anti‐IL‐4R*α* antibody treatment (Table 2B and Figure 4A). In contrast, at the 1:1 (50 ng/mL; w0−w16, *p* < .05) and the 1:10 (10 ng/mL; w0−w16, *p* < .001) allergen dilutions, a significant increase of the basophil reactivity was found (Table 2B and Figure 4B,C). At the 1:100 (1 ng/mL) allergen dilution no statistical differences were detected (Table 2B and Figure 4D).

In patients with grass senitization (rPhl p1 and rPhl p5) that received an anti‐IL‐4R*α* treatment, an increase of the basophil activation to the grass pollen allergen was observable within 16 weeks depending on the allergen concentration. At the 1 (100 ng/mL) and the 1:1 (50 ng/mL) allergen dilutions, no significant changes were observed (Table 2C and Figure 4E,F). In contrast, at the 1:10 (10 ng/mL; w0−w16, *p* < .01) and the 1:100 (1 ng/mL; w0−w16, *p* < .01) allergen dilutions, a significant increase of the basophil activation was detected (Table 2C and Figure 4G,H).

On the other hand, the basophil activation decreased in patients treated with an anti‐IL‐4R*α* antibody after a basophil stimulation with fMLP (positive control; w0−w16: *p* < .05; Table 2D and Figure 4I), but increased after the allergen stimulation. Additionally, basophils displayed an increasing tendency of activation after a stimulation by Fc*ε*RI (positive control), but the observed effect was not determined as statistically significant (Table 2D and Figure 4J).

Since a significant increase of the basophil activation after the allergen stimulation could be observed, we hypothesized that through an IL‐4R*α* blockade induced by a monoclonal anti‐IL‐4R*α* antibody, the activation of basophils were modified depending of the activating stimulus.

Additionally, we calculated the CD‐sens based on a dose‐response curve. In patients receiving the monoclonal anti‐IL‐4R*α* antibody and being sensitized toward birch pollen, the EC50 value decreased (w0−w16, *p* < .05; Table 2B) and, correspondingly, the CD‐sens increased significantly after 16 weeks of treatment (w0−w16, *p* < .05; Table 2B and Figure 4K). A significant decrease of the EC50 from week 4 onward (w0−w16, *p* < .001; w4−w16, *p* < .01; Table 2C) and, correspondingly, a significant increase of the CD‐sens was identified in patients with grass pollen senitization that were treated with a monoclonal anti‐IL‐4R*α* antibody (w0−w16, *p* < .001; w4−w16, *p* < .01; Table 2C and Figure 4L).

**Figure 5:**
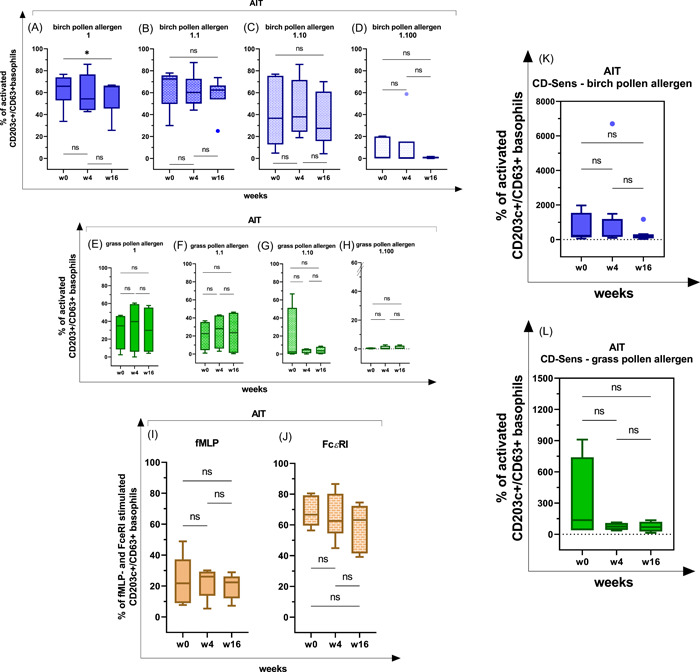
Basophil reactivity and sensitivity in AD patients with a senitization for birch‐ and grass pollen, receiving an AIT; BAT was performed with four different allergen concentrations (1 = 100 ng/mL, 1:1 = 50 ng/mL, 1:10 = 10 ng/mL, 1:100 = 1 ng/mL) for three timepoints [before therapy (w0) and at week 4 (w4) and week 16 (w16)]; ns nonsignificant; *p < .05; **p < .01; ***p < .001; ****p < .0001. In the box‐whisker plots the median, 25th and 75th percentile, the range and outliers are represented. (A–D) Birch pollen specific stimulation: *n* = 7, dilution level 1 (100 ng/mL): *p* (w0–w16) <.05; dilution level 1:1 (50 ng/mL): *p* = ns; dilution level 1:10 (10 ng/mL): *p* = ns; dilution level 1:100 (1 ng/mL): *p* = ns. (E–H) grass pollen specific stimulation: *n* = 4, dilution level 1 (100 ng/mL): *p* = ns; dilution level 1:1 (50 ng/mL): *p* = ns; dilution level 1:10 (10 ng/mL): *p* = ns; dilution level 1:100 (1 ng/mL): *p* = ns. (I) fMLP‐mediated stimulation: *n* = 11, *p* = ns. (J) Fc*ε*RI‐mediated stimulation: *n* = 11, *p* = ns. (K) CD‐sens in birch pollen stimulated CD203c+/CD63+ basophils, *n* = 7, *p* = ns. (L) CD‐sens in grass pollen stimulated CD203c+/CD63+ basophils, *n* = 4, *p* = ns.

In patients receiving an AIT, a significant decrease of the basophil activation after stimulation with the birch pollen allergen was observed at week 16 at the allergen dilution 1 (100 ng/mL; w0−w16, *p* < .05; Table 2I and Figure 5A).

At the 1:1 (50 ng/mL), 1:10 (10 ng/mL) and 1:100 (1 ng/mL) allergen dilutions, no significant effects on the basophil activation were identified (Table 2I and Figure 5B−D).

In patients receiving an AIT and being sensitized to the grass pollen allergen, no significant differences in basophil activation were observed within 16 weeks (Table 2J and Figure 5E−H).

On the other hand, the basophil activation did not change significantly in patients receiving an AIT after a basophil stimulation with fMLP and Fc*ε*RI (Table 2K and Figure 5I,J). Due to the fact that the basophil activation did not change significantly in response to the fMLP‐ and the IgE‐mediated stimulation, but decreased after the birch pollen allergen stimulation, the suppressive effect of AIT was considered to be allergen‐specific.

In AIT‐patients with birch and grass pollen senitization, a decreasing tendency of the CD‐sens was observable, but due to the small number of patients recruited, the effect was not statistically significant (Table 2I,J and Figure 5K,L).

Overall, an IL‐4R*α* blockade by a monoclonal anti‐IL‐4R*α* antibody significantly increased the basophil reactivity and the basophil sensitivity depending on the activating stimulus, whereas the AIT decreased the basophil responsiveness.

### 3.3 T cell proliferation in response to seasonal allergens was found decreased in patients treated with an anti‐IL‐4R*α* antibody or with an AIT

To investigate the effect of the IL‐4R*α* blockade through the administration of a monoclonal anti‐IL‐4R*α* antibody versus that of an AIT on specific T cells deriving from AD patients with comorbid ARC, we analysed the proliferative response in parallel to the basophil activation and the sIgE levels.

The proliferation in response to the birch‐induced stimulation decreased significantly in CFSE‐treated CD4+ cells deriving from PBMCs of patients treated with a monoclonal anti‐IL‐4R*α* antibody or with AIT, depending on the allergen concentration.

**Figure 6:**
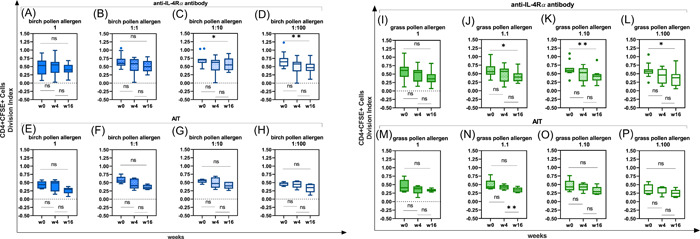
T cell proliferation under an anti‐IL‐4R*α* antibody or AIT. Comparative analysis of the median response for the variable division‐index according to birch‐and grass pollen stimulation (1 = 100 ng/mL, 1:1 = 50 ng/mL, 1:10 = 10 ng/mL, 1:100 = 1 ng/mL) in two therapy groups and three observational points (w0, w4, w16); ns nonsignificant; *p < .05; **p < .01; ***p < .001; ****p < .0001. The Global (Min‐Max) and Interquartile (25%–75%) ranges are shown. (A–H) Frequency of CFSE+ CD4+ after 7 days of cell culture after birch pollen stimulation in patients with an anti‐IL‐4R*α* antibody (A–D, *n* = 11) dilution level 1 (100 ng/mL): *p* = ns; dilution level 1:1 (50 ng/mL) *p* = ns; dilution level 1:10 (10 ng/mL): *p* (w0–w16) <.05; dilution level 1:100 (1 ng/mL): *p* (w0–w16) <.01, and AIT (E–H, *n* = 5). (I–P) Frequency of CFSE+ CD4+ after 7 days of cell culture after grass pollen stimulation in patients with an anti‐IL‐4R*α* antibody (I–L, *n* = 11) dilution level 1 (100 ng/mL): *p* = ns; dilution level 1:1 (50 ng/mL) *p* (w0–w16) <.05; dilution level 1:10 (10 ng/mL): *p* (w0–w16) <0.01; dilution level 1:100 (1 ng/mL): *p* (w0–w16) <.05 and AIT (M–P, *n* = 5) dilution 1:1 (50 ng/mL) *p* (w4–w16) <0.01.

In patients treated with a monoclonal anti‐IL‐4R*α* antibody, no significant differences in T cell proliferation were found after a birch‐induced stimulation at the 1 (100 ng/mL) and the 1:1 (50 ng/mL) allergen dilutions (Table 2E and Figure 6A,B), but this was not the case for the 1:10 (10 ng/mL; w0−w16, *p* < .05) and the 1:100 (1 ng/mL; w0−w16, *p* < .01) allergen dilutions after 16 weeks (Table 2E and Figure 6C,D).

In contrast, the T cells of AIT‐treated patients did not exhibit any differences in terms of their proliferation after a birch‐induced stimulation from week 4 onward (Table 2K and Figure 6E−H).

As far as the grass pollen allergen is concerned, T cells did not show any significant diminished division at allergen dilution 1 (100 ng/mL) within 16 weeks after an anti‐IL‐4R*α* antibody treatment (Table 2F and Figure 6I), but this was not the case at the 1:1 (50 ng/mL; w0−w16, *p* < .05), 1:10 (10 ng/mL; w0−w16, *p* < .01) and 1:100 (1 ng/mL; w0−w16, *p* < .05) allergen dilutions (Table 2F and Figure 6J−L).

In AIT‐treated patients the CFSE‐treated CD4+ cells decreased significantly from week 4 onward after a grass pollen allergen stimulation. A significant decrease in T cell proliferation was observed at the 1:1 (50 ng/mL) dilution (w4−w16, *p* < .01; Table 2M and Figure 6M,N). No significant changes in T cell proliferation were found at the 1 (100 ng/mL), the 1:10 (10 ng/mL) and the 1:100 (1 ng/mL) allergen dilutions tested (Table 2M and Figure 6O,P).

No statistical differences were observed with regard to the T cell proliferative indexes response after a birch‐ and a grass‐induced stimulation with human recombinant IL‐2 (Figure S2) and in cell stimulation positive controls with monoclonal anti‐biotin human antibodies against CD2, CD3 and CD28, recombinant human IL‐2 or RPMI. In summary, the anti‐IL‐4R*α* antibody treatment, as well as the AIT decreased the allergen‐specific T cell proliferation.

### 3.4 ARC symptoms

In patients treated with an anti‐IL‐4R*α* antibody, the severity of ARC symptoms decreased significantly within 4 weeks, whereas no significant changes were observed in patients under an AIT.

In addition to the measurement of total IgE‐ and sIgE levels, we examined the severity of ARC symptoms based on the RCAT. In patients receiving an anti‐IL‐4R*α* antibody, the ARC symptoms improved significantly (w4) within 4 weeks (Table 2G). In contrast, in patients receiving an AIT, no significant changes of the allergic symptoms were observed within the observational time of 16 weeks (Table 2N).

## 4 DISCUSSION

Patients with AD and comorbid ARC under therapy with a monoclonal anti‐IL‐4R*α* antibody (index group) or receiving an AIT (control group) were, herein, studied to investigate how the monoclonal anti‐IL‐4R*α* antibody impacts the total IgE and the sIgE levels as well as the allergen‐specific basophil activation and T cell proliferation. Our findings indicate a significant decrease of the total IgE and the sIgE serum levels and of the T cell response, but also a significant increase of the basophil activation and sensitivity within 16 weeks in patients treated with the monoclonal anti‐IL‐4R*α* antibody. In patients receiving an AIT, the allergen‐specific basophil activation and the T cell proliferation in response to seasonal allergens were found to be significantly decreased.

First, we found reduced total IgE and sIgE levels in patients treated with a monoclonal anti‐IL‐4R*α* antibody, but not in patients receiving an AIT. While the anti‐IL‐4R*α* antibody inhibits the IL‐4‐mediated pathways such as the Th2‐differentiation and B cell induction, a reduced B cell induction can cause a reduced IgE synthesis and, therefore, might explain the reduced IgE levels.^37^, ^38^ In patients receiving an AIT, their IgE levels did not change significantly during the observational period of 16 weeks, whereas significantly decreased IgE levels were detected at later timepoints in previous studies on AD patients receiving an AIT.^58^, ^59^

In addition to the measurement of the total IgE and the sIgE levels, we examined the patients' ARC symptoms based on the RCAT. In patients treated with a monoclonal anti‐IL‐4R*α* antibody, a significant reduction of their ARC symptoms was observed, but this was not the case in patients receiving an AIT. The significant reduction of the ARC symptoms as a result of the anti‐IL‐4R*α* antibody treatment is in line with the reduced total IgE and sIgE levels that we observed in our study. In patients receiving an AIT, their ARC symptoms did not change significantly during the observational period of 16 weeks. In previous studies significant changes in the ARC symptoms as a result of an AIT were detected at later times, taking into account that an AIT can last at least 3 years.^27^, ^59^

Second, the allergen‐specific basophil activation and sensitivity exhibited a decreasing tendency in patients under a birch‐specific AIT; however, in patients treated with the monoclonal anti‐IL‐4R*α* antibody, a significant increase of the basophil responsiveness was observed. Several studies have reported a suppressive effect of AIT on basophil activation ^41^, ^42^, ^43^, ^44^ and have indicated this as a criterion of the treatment's efficacy.^40^

On the other hand, we observed a significant increase of the basophil activation and sensitivity within 16 weeks under a monoclonal anti‐IL‐4R*α* antibody therapy. It might be possible that the increased basophil activation response to the anti‐IL‐4R*α* antibody is associated with a feedback mechanism, in which the basophils overreact to the reduced IgE‐mediated stimulation caused by the lower B cell induction. There are reports in which the briefly increasing effects of basophils are described after the beginning of a treatment with the IgE‐blocking monoclonal antibody omalizumab in patients with chronic spontaneous urticaria (CSU).^60^, ^61^, ^62^ Alizadeh Aghdam et al.^61^ examined the effects of different stimulus‐secretion pathways of basophil granulocytes and described an upregulation of the IgE‐mediated pathway by Fc*ε*RI, whereas the IgE‐independent pathway via G‐protein‐coupled C5a receptors was down regulated during IgE‐blocking by Omalizumab.

In our study two activation routes of basophil granulocytes were tested at the beginning of the BAT using two positive controls (IgE‐dependent via Fc*ε*RI vs. IgE‐independent via fMLP). We observed a significant decrease of basophil activation in patients treated with an anti‐IL‐4R*α* antibody after a basophil stimulation with fMLP within 16 weeks. Additionally, basophils displayed no significant changes after a stimulation via Fc*ε*RI without an allergen‐specific stimulus. However, after allergen‐specific stimulation with birch‐ and grass pollen allergen, a significant increase of the basophil activation was observable under an IL‐4R*α* blockade.

We assumed that through an IL‐4R*α* blockade induced by a monoclonal anti‐IL‐4R*α* antibody, the IgE‐independent reaction pathway via fMLP has been down regulated, whereas the allergen‐specific activation of basophils were mediated via the IgE‐dependent pathway and modified depending of the activating stimulus.

Another report has described the effect of an increased basophil sensitivity in CSU patients treated with omalizumab as “reversible,” because 3 months after the termination of the IgE‐blocking therapy, the basophil sensitivity returned to baseline levels.^62^ It might be possible, that the increased basophil reactivity and sensitivity observed in our study also decreases during the treatment with the monoclonal anti‐IL‐4R*α* antibody. In future studies, it would be useful to monitor the parameters for longer periods of time to investigate the process of the basophil responsiveness in self‐terminating cases and in desensitization pathways.^63^

To examine the biological efficacy of a monoclonal anti‐IL‐4R*α* antibody in AD patients with comorbid ARC, we followed the T cell proliferative response after an in vitro allergen‐specific stimulation. Our findings revealed a significant decrease in CFSE‐treated CD4+ cells from baseline through week 16 under the anti‐IL‐4R*α* antibody treatment. Our data are in line with those of a previous report that has demonstrated a functional blockade of the IL‐4R*α* in T cells from week 4 through week 52 in patients with moderate to severe AD.^64^ Remarkably, our study confirmed the inhibitory effect of the anti‐IL‐4R*α* antibody on the ex vivo proliferative response of T cells deriving from PBMCs after a 7‐day allergen‐specific stimulation. Some reports have emphasized the role of the IL‐4R*α* pathway and its association in modulating immune tolerance by undermining allergen‐specific Treg cell responses.^17^ The blocking of the IL‐4R*α* pathway might be crucial for the induction of allergen‐specific Tregs and the reversal of the allergic inflammatory response. Notwithstanding, some reports suggest that the IL‐4R*α*‐mediated signaling pathway can induce Forkhead‐box‐P3 + Treg cells to optimally control Th2 inflammation during infections associated with a Th2 response, such as those caused by helminths.^65^ Similarly, it has been reported that the IL‐4R*α* binding with IL‐4 and IL‐13 is important for the generation of functional Tregs in an antigen‐dependent manner.^66^

### 4.1 Strengths and limitations

Our study is the first attempting to analyse the impact of a monoclonal anti‐IL‐4R*α* antibody on basophil activation and allergen‐specific T cell proliferation in vitro, in patients with AD and comorbid ARC. We have, herein, selected the patients according to their sIgE levels (being ≥5 kU/L) and/or their ARC symptoms (based on the RCAT being ≤17) to assure a symptomatic comorbid ARC. We have also monitored the development of the parameters for 16 weeks, based on data from the registration studies of the monoclonal anti‐IL‐4R*α* antibody.^18^ Moreover, significant changes could be indicated more easily at the beginning of the treatment, without the interference of disruptive factors or external triggers. We have chosen AD patients with AIT‐treated ARC as a control group, because the effects of AIT on IgE levels and the basophil activation have been described in several studies and are, therefore, well known.^40^, ^43^, ^44^, ^45^ Furthermore, our patients filled in a questionnaire based on the DLQI as well as the RCAT and the EASI; this questionnaire enabled us to evaluate the efficacy of the monoclonal anti‐IL‐4R*α* antibody and to ensure the correct handling, the detection of nonresponders and the minimizing of bias.

A limitational factor in our study was the fact that most of the patients were polysensitised and, therefore, the effects of external allergen triggers could not be excluded. Additionally, the observational time was only 16 weeks, but the treatment with a monoclonal anti‐IL‐4R*α* antibody lasts longer in patients with AD, while the administration of an AIT should be conducted for a total of 3 years. Furthermore, the study took place during the COVID‐19 pandemic and the recruitment of the patients had to be halted; as a result, only a small number of patients receiving an AIT were finally included in our study.

## 5 CONCLUSION

The purpose of our study was to investigate the effect of a monoclonal anti‐IL‐4R*α* antibody on the in vitro allergic response of basophils and T cells deriving from AD patients with comorbid ARC. We have, herein, described a trend of increasing basophil activity and sensitivity under a treatment with a monoclonal anti‐IL‐4R*α* antibody, whereas the patients' serum IgE levels and their T cell proliferation decreased significantly. Nonetheless, long‐term effects were not evaluated by our study and might be part of future research projects.

## AUTHOR CONTRIBUTIONS

**Anne‐Sophie Layritz**: formal analysis; investigation; methodology; project administration; visualization; writing—original draft; writing—review & editing. **Jorge Galicia‐Carreón**: formal analysis; investigation; methodology; visualization; writing—review & editing. **Said Benfadal**: Investigation. **Natalija Novak**: conceptualization; funding acquisition; project administration; supervision; validation; writing—review & editing.

## CONFLICT OF INTEREST STATEMENT

Natalija Novak reports grants and personal fees from Abbvie, Allergopharma, Alk Abello, Bencard Allergy Therapeutics, Blueprint, HAL Allergy, Leti Pharma, Leo Pharma, Eli Lilly, Lofarma, Novartis, Pfizer, Regeneron, Sanofi Genzyme, Stallergenes‐Greers, Streamed up, Thermo Fisher. The rest of the authors declare no conflicts of interest.

## ETHICS STATEMENT

This study protocol was reviewed and approved by the Ethics committee of the University Hospital Bonn; approval number 267/19. All donors gave their written informed consent before starting the study.

## Supporting information

Supplementary information.

Supplementary information.

## Data Availability

The data that support the findings of this study are available from the corresponding author upon reasonable request.
