# Supplementary material for: Differences in allergen‐specific basophil activation and T cell proliferation in atopic dermatitis patients with comorbid allergic rhinoconjunctivitis treated with a monoclonal anti‐IL‐4Rα antibody or allergen‐specific immunotherapy
Source: Immun Inflamm Dis. 2023 Apr 12;11(4):e808. doi: 10.1002/iid3.808 (PMC10091378; doi:10.1002/iid3.808)
Supplement: Supplementary file 1 — Supplementary information. [file IID3-11-e808-s001.pdf]

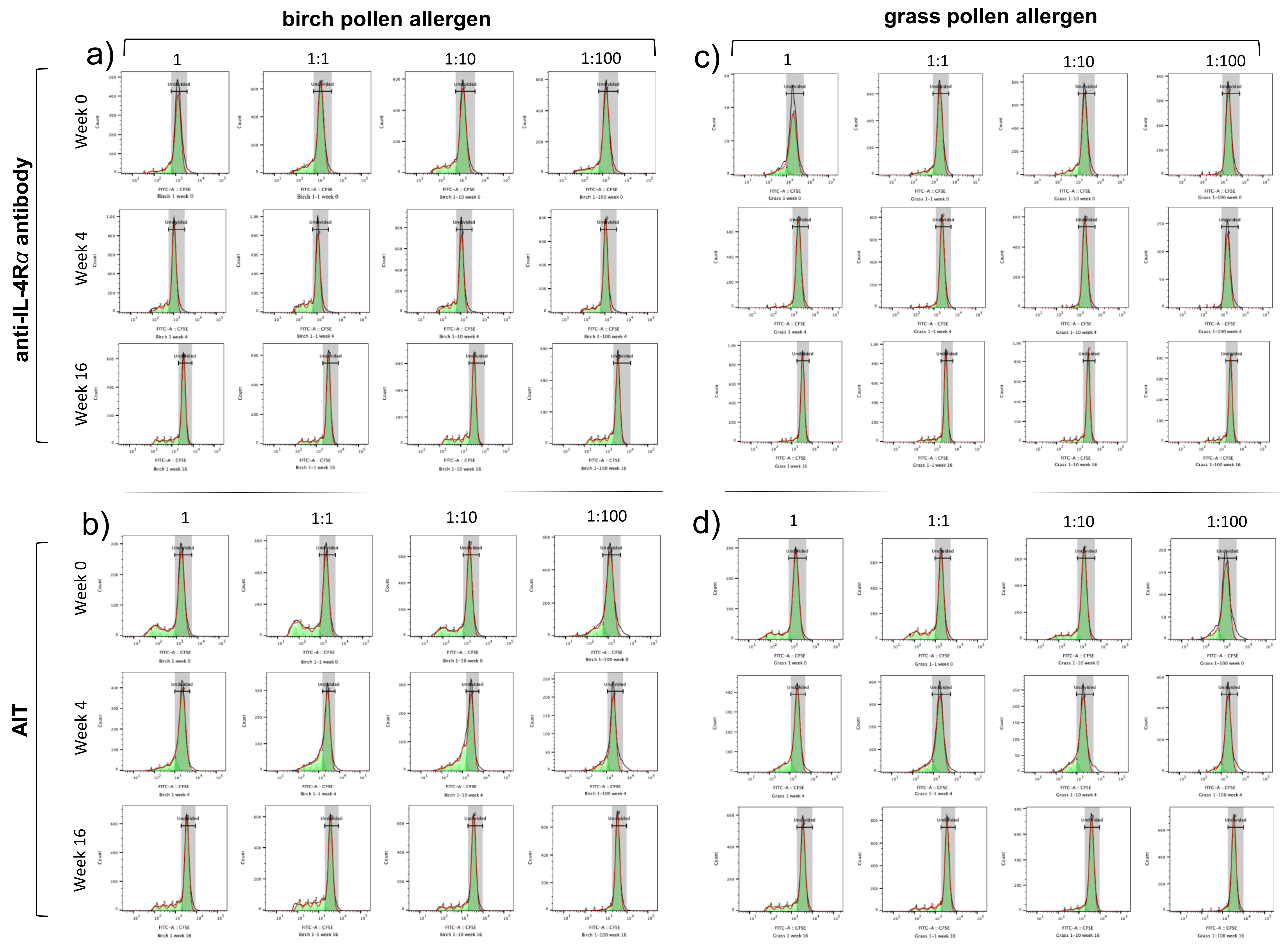

<u>Fig. S1:</u> Representative histograms showing a progressive dilution in the CFSE fluorescent intensity of proliferative T cells for anti-IL-4R*α* antibody (a,c) and AIT (b,d) in AD patients. The fluorescence of the stimulated CD4+ CFSE-diluted cells was measured after 10 days of allergen-stimulation with birch- (a,b) and grass pollen allergen (c,d) in diluted concentrations (1 = 100 ng/ml, 1:1 = 50 ng/ml, 1:10 = 10 ng/ml and 1:100 = 1 ng/ml) and human recombinant IL-2 (50-100 UI/ml, every 2 days) at week 0 (w0), 4 (w4) and 16 (w16) respectively.

Figure_S1_Layritz;Galicia et al.
