# Supplementary material for: Differences in allergen‐specific basophil activation and T cell proliferation in atopic dermatitis patients with comorbid allergic rhinoconjunctivitis treated with a monoclonal anti‐IL‐4Rα antibody or allergen‐specific immunotherapy
Source: Immun Inflamm Dis. 2023 Apr 12;11(4):e808. doi: 10.1002/iid3.808 (PMC10091378; doi:10.1002/iid3.808)
Supplement: Supplementary file 2 — Supplementary information. [file IID3-11-e808-s002.pdf]

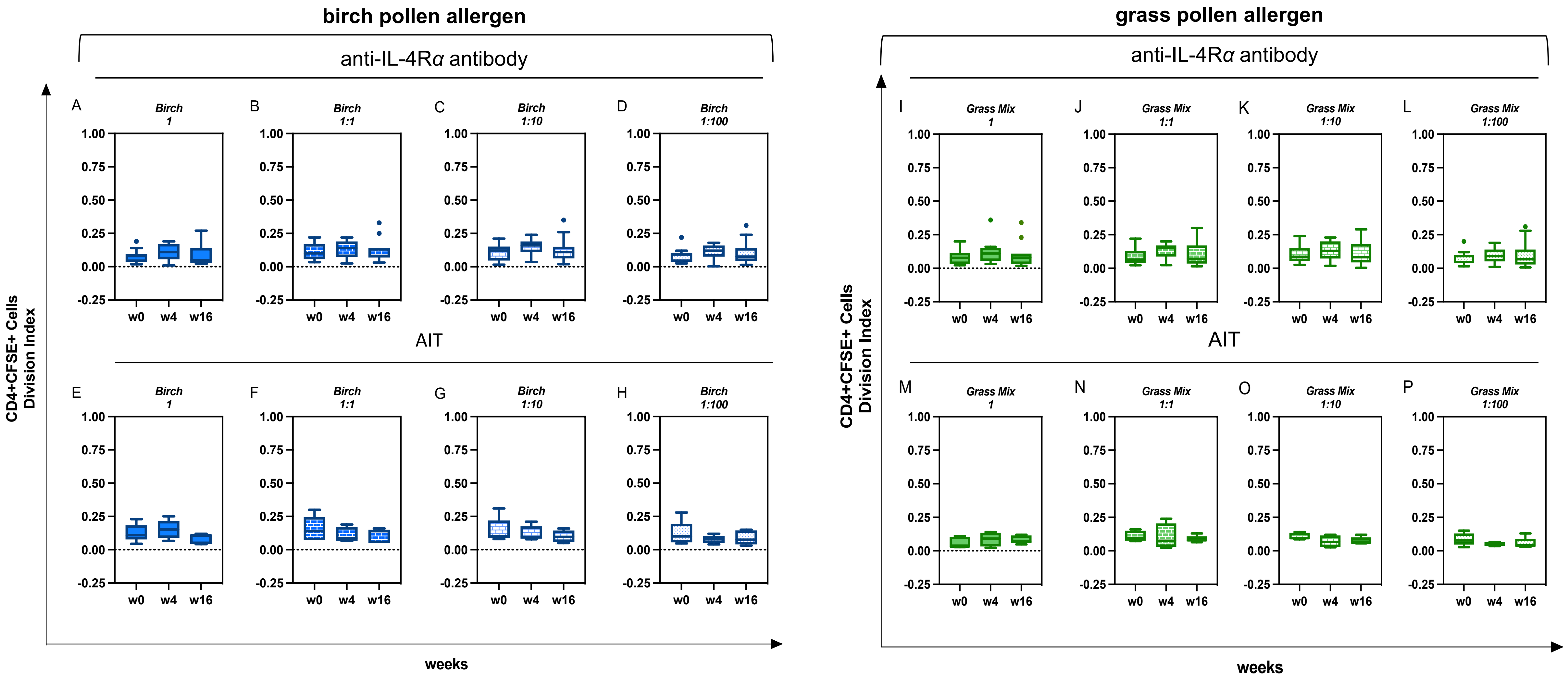

<u>Fig. S2:</u> T cell proliferation under an anti-IL-4Rα antibody or AIT; Comparative analysis of the median response for the variable division-index according to birch- and grass pollen stimulation (1 = 100 ng/ml, 1:1 = 50 ng/ml, 1:10 = 10 ng/ml and 1:100 = 1 ng/ml) and human recombinant IL-2 (50-100 UI/ml, every 2 days) in two therapy groups and three observational points (w0, w4, w16); ns non-significant; * p<0.05; ** p<0.01; *** p<0.001; **** p<0.0001. The Global (Min-Max) and Interquartile (25%-75%) ranges are shown.

(A-H) Frequency of CFSE+CD4+ after 10 days of cell culture after birch pollen stimulation in patients treated with an anti-IL-4Rα antibody (A-D, n=11) dilution level 1 (100 ng/ml): p=ns; dilution level 1:1 (50 ng/ml) p=ns; dilution level 1:10 (10 ng/ml): p=ns; dilution level 1:100 (1 ng/ml): p=ns, and AIT (E-H, n=5): p=ns.

(I-P) Frequency of CFSE+CD4+ after 10 days of cell culture after grass pollen stimulation in patients treated with an anti-IL-4Rα antibody (I-L, n=11) dilution level 1(100 ng/ml): p=ns; dilution level 1:1 (50 ng/ml) p=ns; dilution level 1:10 (10 ng/ml): p=ns; dilution level 1:100 (1 ng/ml): p=ns and AIT (M-P, n=5): p=ns.

figure _S2_Layritz; Galicia et al.
